# Developments in resin-based composites

**DOI:** 10.1038/s41415-022-4240-8

**Published:** 2022-05-13

**Authors:** Matthew J. German

**Affiliations:** School of Dental Sciences, Translational and Clinical Research Institute, Newcastle University, Framlington Place, Newcastle upon Tyne, UK

## Abstract

With the phasing down of dental amalgam use in response to the Minamata Convention, it is likely that resin-based composite restoratives will be the dental material of choice for the direct restoration of compromised dentition in the UK, at least for the foreseeable future. The current materials have a finite lifespan, with failures predominately due to either secondary caries or fracture. Consequently, there is considerable *in vitro* research reported each year with the intention of producing improved materials. This review describes the recent research in materials designed to have low polymerisation shrinkage and increased mechanical properties. Also described is research into materials that are either antimicrobial or are designed to release ions into the surrounding oral environment, with the aim of stimulating remineralisation of the surrounding dental tissues. It is hoped that by describing this recent research, clinicians will be able to gain some understanding of the current research that will potentially lead to new products that they can use to improve patient treatment in the future.

## Key points

- Provides an overview of recent research developments aimed at improving the performance of resin-based composites.
- Details the recent developments in monomers and fillers to produce resin-based composites that either have lower polymerisation shrinkage or better mechanical properties compared to current commercially available products.
- Describes recent research on developing resin-based composites that can act as potential sources of antimicrobial or remineralising agents.

## Introduction

With the phasing out of dental amalgams, resin-based composite (RBC) restoratives will be the dental material of choice for the direct restoration of compromised dentition in the UK, at least for the foreseeable future.^1^^,^^2^ With much better colour-matching to the surrounding dentition and more conservative cavity preparation typically required for RBCs compared with dental amalgams,^3^ they are already a popular choice for many practitioners worldwide.^4^^,^^5^ Despite this, the UK is still an area of high dental amalgam use, particularly in the publicly funded sector.^6^^,^^7^ The best results with RBCs are obtained when using rubber dam and acid-etch bonding,^8^ which can increase treatment times; clinicians worry that the extra time and expense involved means that the NHS will have to modify the fee payment structure if dental amalgam is replaced.^6^ Despite dental amalgams and RBCs apparently performing equally well in small and large load-bearing restorations,^4^^,^^9^ there remain concerns that RBCs have a relatively shorter lifespan than dental amalgams and many UK clinicians report a lack of confidence in using RBCs, compared to dental amalgams, for use in complicated procedures.^10^ When RBC failures occur, they are mostly due to secondary caries or fracture.^2^^,^^11^ Consequently, most laboratory RBC research tends to focus on trying to make improvements to combat one or both of these. This review is intended to bring together some of the main themes in this research, with a view to offer the practising clinician some indication as to how the current research may lead to improved RBCs in the future.

## Brief history

The historical development of RBCs has been comprehensively summarised previously.^11^^,^^12^ Briefly, the major developments in RBCs can be most conveniently divided into developments in the monomers, the fillers and the initiators. It is often considered that the development of the higher molecular weight difunctional monomer, bisphenol A-glycidyl methacrylate (BisGMA) by Bowen in the early 1960s started the development of modern RBCs. This high molecular weight led to a reduced polymerisation shrinkage compared to acrylic resins. Additionally, the stronger monomer backbone and crosslinking during polymerisation gave improved mechanical properties in the finished restoration. However, BisGMA is highly viscous, meaning diluent monomers such as triethylene glycol dimethacrylate were needed to lower viscosity, which meant manipulation of the RBC was easier and higher filler loadings could be achieved. Subsequently, other monomers such as urethane dimethacrylate and ethoxylated bisphenol-A dimethacrylate were developed, but BisGMA's superior mechanical properties mean that most currently available RBCs contain at least some BisGMA.

Higher concentrations of filler led to improved mechanical properties and lower polymerisation shrinkage, in general as a result of a reduced monomeric resin constituent. However, different approaches were taken to achieve higher filler loadings leading to different classes of RBCs being developed, such as microfills and hybrids. Particle sizes were reduced to the sub-micron scale, long before the advent of the so-called nanocomposites. There was often similarity in the particle sizes used for the filler in these classes, with the differences in products really based on the filler concentration and how the fillers were produced and incorporated in the resin matrix. Consequently, clinicians had a variety of composite classes they could use to restore a variety of conditions, with the RBC classes based on the manufacturers' marketing strategy, rather than a scientific analyses of the resultant properties.^13^^,^^14^

The initially used chemically activated polymerisation took longer than patients or clinicians desired and so photoinitiators were added. First, ultraviolet-sensitive photoinitiators were used, but they produced limited depth of cure (DOC) and degree of conversion (DC%), so visible-light-sensitive photoinitiators, such as camphorquinone (CQ), were introduced. To activate the photoinitiation, light-curing units (LCUs) capable of delivering light at the correct excitation wavelengths were developed, first using broadband sources such as quartz-tungsten-halogen bulbs and more recently, narrowband light-emitting diode (LED) sources tuned to the specific excitation wavelengths. With improvements in many properties linked to the DC%, increasingly intense light sources have been developed with the aim of increasing the DC%. However, the relationship between DC% and light intensity is complicated and it has been shown the once the LCU intensity increases above 1 W/cm^2^, any further improvement in properties may be marginal.^15^ These high intensity LCUs are also suggested to reduce the time required to obtain a satisfactory DC% because, it is claimed by manufacturers, that there is a simple reciprocity relationship between light intensity and curing time. However, investigation using both commercially available RBCs^16^ and laboratory-produced model formulations,^17^ different types of LCUs^18^and photoinitiators,^17^ have revealed that the relationship is more complex and factors such as overall monomer viscosity, monomer types used and filler concentration are all important. In general, it seems that so long as the radiant exposure from the LCU is above the minimum required to produce adequate polymerisation, the reciprocity relationship holds so long as the filler loading is above a 50 wt%.^19^

## Low shrink RBC materials

The main reason for RBC replacement is due to dental caries, either a recurrence of the original caries, or secondary caries.^5^ Although there is still no direct clinical evidence to prove that polymerisation shrinkage is the cause of secondary caries,^5^ *in vitro* studies show that it can cause cuspal deflection, enamel cracking and the breakdown of the composite-tooth margin,^5^^,^^20^^,^^21^^,^^22^ the latter of which could potentially lead to caries and is taken as justification for the considerable amount of research undertaken to develop so-called low shrink materials. Meeries *et al*.^23^ conducted a meta-analysis of much of this work in 2018. The magnitude of the shrinkage strain and stress during polymerisation has many causes and so it is not surprising that many different approaches have been attempted to reduce it.^24^ Broadly speaking, these approaches can be divided into: alterations in the filler size and concentration; and the monomer structure,^23^^,^^24^ although some research has focused on modifying the coupling agent^20^^,^^25^^,^^26^ and altering the polymerisation initiation rate by initially reducing the intensity of light emitting from the LCU.^27^

Increasing the concentration of filler leads to a reduction in polymerisation shrinkage, simply due to the relative reduction in reactive monomer groups per unit volume.^28^ By reducing the size of filler particles and including multiple size distributions into the monomer, filler concentrations have raised to well over the 50 vol%, which was the limit for the first RBCs. One of the limits identified with including higher concentrations of filler was that it became harder for the monomer to wet all the filler particles, meaning there came a point when mechanical properties were reduced with increasing filler concentration. Consequently, smaller nanoscale particles were developed. Traditional methods of making filler particles, such as milling and sieving, tend to be unable to produce particles smaller than of the order of 100 nm.^29^ Consequently, techniques such as pyrolysis and sol-gel production have been utilised.^30^ In general, silica nanoparticles are amorphous and spherical, although as they grow larger, they tend to be less regular in shape.^31^ With such small particles, the ratio of surface energy to volume can be sufficiently high, that particles tend to agglomerate into clusters that can be up to 5 μm in diameter,^32^ which can lead to a poor distribution of filler in an RBC. While some beneficial properties, such as improved wear resistance, have been reported for the agglomerated nanocluster materials,^29^^,^^33^ most often, organosilane coupling agents are used to reduce agglomeration^29^^,^^34^ and improve the properties. By incorporating nanoparticles into multi-particle distribution hybrid systems, increased filler concentrations have been reported with related increases in a variety of mechanical properties^35^^,^^36^ and decreases in polymerisation shrinkage. There is considerable variability in the distribution, concentration and relative amounts of microparticles and nanoparticles in current commercially available nanohybrids,^14^ suggesting that once again, this description of a class of RBC is more akin to a marketing strategy than useful to the clinician when choosing a material for use.

In addition to altering the dimensions and concentration of filler in RBCs to reduce shrinkage, alterations to the monomeric resin constituents are commonplace. This is not surprising, since the monomer is the component responsible for the shrinkage and it has been known for many years that the amount of volumetric shrinkage that occurs during polymerisation is proportional to the molecular weight of the monomer, for methacrylate monomers at least.^37^ A whole variety of monomer families have been reported to produce 'low-shrink' RBCs, ranging from alternative methacrylates,^38^^,^^39^^,^^40^ thiol-enes,^41^ thiol-urethanes^42^ and siloranes.^21^ The *in vitro* data for these different monomers show promising results, with shrinkages significantly lower compared with those obtained with BisGMA RBC derivatives and often with improvements in the mechanical properties. Several commercial products have been released, notably those based on silorane and some dimethacrylates; however, the initially promising *in vitro* data on these materials does not seem to have translated to a clinical advantage since the first commercially available silorane-based RBC formulation was withdrawn from the market.

## Stronger RBC materials

With fracture being the other most common cause of RBC restoration failure, many studies have focused on developing stronger materials. Improvements in fillers have been suggested by developing ceramic and glass-ceramic materials, some of which have already been incorporated in commercially available products. One area that is also receiving considerable attention is attempts to improve the resin component, either by developing stronger monomers or by increasing the degree of conversion.

Employing current LCUs, the conversion of monomer to polymer clinically is typically below 80%, and can be as low as 40%.^43^^,^^44^ As the most common monomers used are difunctional, any degree of conversion above 50% might suggest that each monomer is converted to polymer. However, as many studies have shown that there is residual unreacted monomer that can be released into the oral environment,^44^^,^^45^^,^^46^^,^^47^^,^^48^ a conversion more than 50% is needed. Health concerns remain over the release of monomer into the oral environment due to their potential irritancy and cytotoxicity.^47^ Of particular concern is the bisphenol A (BPA) component of BisGMA. BPA mimics the effects of oestrogen and in animal studies has been shown to be potentially linked to several health conditions, particularly a reduction in fertility^49^ and *in utero* exposure could alter organ development.^50^ In products such as baby drinking bottles, many products are now explicitly advertised as BPA-free and while the link between BisGMA-based RBCs and any of the health problems associated with BPA has never been established, improved monomers could potentially lead to longer lasting restorations and avert another 'amalgam debate'.

Methacrylate monomers, such as those used in RBCs, have ester bonds that are susceptible to hydrolysis and so weaken over time.^51^ Consequently, alternative monomers more stable in aqueous environments are being researched. As with low-shrink monomers, a wide variety of monomer families have been reported, such as diacrylates, methacrylamides, vinyl ethers, thiol-vinyl sulphone and thiol-enes.^52^^,^^53^ In some cases, different polymerisation routes, such as step-growth polymerisation,^54^ click-chemistry^55^ and reversible addition-fragmentation chain transfer^56^ have been used. Initial *in vitro* data for composites made from these monomers seem promising, with high degrees of polymerisation and increased mechanical properties reported compared to currently available RBC products.

The most common photoinitiator used in RBCs is CQ, a Norrish type II initiator, which requires a co-initiator, such as a tertiary amine, to generate a sufficiently high concentration of radicals.^57^ Most *in vitro* research tends to use as the co-initiator either (dimethylamino)ethyl methacrylate (DMAEMA) or ethyl-4-(dimethylamino) benzoate (EDAB),^58^ with EDAB typically reported to be the most efficient of the two.^58^^,^^59^ However, the desire to have higher DC% and faster polymerisation has led to other initiators being investigated. The addition of iodonium salts as co-initiators has been shown to increase the rate of polymerisation, DC% and mechanical properties of CQ/amine systems due to the iodonium salts increasing the number of radicals produced per CQ molecule.^58^^,^^60^^,^^61^^,^^62^^,^^63^ These encouraging results using iodonium salts has even led researchers to consider whether amine-free systems are possible, but so far, the properties of amine-free RBCs are below those containing CQ/amine/iodonium salts.^64^

Norrish type I initiators, such as derivatives of acylphosphine oxides and of benzoyl germanium, have been also been considered.^65^ These form radicals by a cleavage reaction and so do not need a co-initiator.^57^ They also tend to have a less obvious effect on the colour of RBCs compared to CQ.^59^^,^^66^ One benzoyl germanium derivative, bis-(4-methoxybenzoyl)diethylgermane, has already been patented under the trademark Ivocerin and is used in commercially available products.^67^ The acylphosphine oxides are widely researched, typically providing much greater rates of polymerisation and higher DC%^68^ but with lower DOC.^66^ They also have excitation wavelengths different to that of CQ, meaning that for optimal polymerisation, different LCUs are needed. Many manufacturers now market 'polywave' LCUs that contain multiple LEDs capable of delivering light at different wavelengths, but this represents an increased cost for clinicians if they are to use RBCs that contain these alternative initiators. When type I and type II initiators are combined, improvements in DOC and colour stability have been reported compared relative to type I (DOC) and type II (colour stability) only systems.^66^^,^^68^ In the search for stronger composites, it is likely that more research will focus on combinations of photoinitiators, particularly now that polywave LCUs are readily available.

## Functional RBCs

Current RBCs act really only as a space filler, returning form and function to the surrounding tooth, yet other filling materials are known to have an antibacterial effect, for instance low copper dental amalgams and to release fluoride, for instance glass ionomer cements. As RBCs are developed it is not, therefore, surprising that researchers have attempted to add these types of capabilities to them.

RBCs that have a bactericidal effect have been developed with the twin aims of either killing any residual bacteria that remain after cavity preparation and/or to reduce the incidence of secondary caries. RBCs that can release ions of silver or zinc are well-known to show antibacterial action *in vitro*.^69^^,^^70^ Others have been modified to contain commonly used soluble antibacterial agents such as chlorhexidine (CHX). As the agents are not bound to the RBC, they wash out at initially high concentrations that diminishes rapidly over time, typical of a diffusion controlled release profile,^71^ which also compromises the mechanical properties of the RBC.^72^ Methacrylate monomers functionalised with agents such as CHX have been developed^73^ with the aim of extending the antibacterial activity beyond the initial burst period. While the CHX-methacrylates have been used in experimental RBCs and are already included in some commercially available dentine bonding agents, far more commonly used in RBC research are quaternary ammonium compounds. Many different quaternary ammonium methacrylates (QAMs) have been studied and they have demonstrated antibacterial activity against single species models and multi-species models, including bacteria obtained directly from saliva or dental plaque.^74^ One QAM, methacryloyloxydodecylpyridinium bromide (MDPB) has been incorporated into model RBCs, with some encouraging *in vitro* results^75^^,^^76^^,^^77^^,^^78^ and has been a component of a commercially available dentine bonding agent for some time. The results of *in vitro* and *in situ* studies using the DBA are encouraging and suggest that QAM-containing materials may well reduce bacterial adhesion.^79^ QAMs work by a contact killing mechanism, meaning that there are some concerns that their antibacterial action will diminish once the RBC surface is covered by pellicle.^80^^,^^81^ Consequently, QAMs have been combined with zwitterionic monomers to stop the pellicle from forming.^82^ Zwitterions, such as 2methacryloyloxyethyl phosphorylcholine (MPC), have two oppositely charged groups in their structure and are highly hydrophilic, making it difficult for proteins and bacteria to adhere to them.^83^ In a recent *in vitro* study, these combined MDPB/MPC materials exhibited promising antibacterial activity and protein repulsion, although it should be noted that these effects were only studied over a 48 hour period.^82^

A variety of salts, ceramics and glasses have been developed to act as potential fillers for RBCs that release ions such as calcium, fluoride and phosphate when exposed to an aqueous environment. The role of fluoride ions in the prevention of caries when used in gels and mouthwashes is well-documented.^84^ It is hoped ion-releasing fillers will act in a similar way, but depending on the filler composition, also stimulate the formation of either hydroxyapatite or fluorapatite, in the surrounding enamel and dentine. Of particular interest are fillers based on calcium phosphates,^85^^,^^86^^,^^87^^,^^88^ calcium fluoride,^86^ fluorapatite^89^^,^^90^^,^^91^ and a variety of glasses based on SiO_2_-P_2_O_5_-CaO-Na_2_O (termed BioGlasses),^92^ recently reviewed.^93^ *In vitro* assessment of RBCs containing these fillers has revealed they can form apatite layers^94^^,^^95^ when exposed to simulated body fluid (SBF).^96^^,^^97^^,^^98^ However, SBF does not mimic the organic components of saliva, raising some concerns that the *in vitro* apatite formation may not be replicated *in vivo*, so some caution must be exercised when interpreting these results.^99^ The majority of current studies involve modifications to the composition of the filler, the method of producing the filler and alteration of filler concentration, meaning that clear structure-property relationships do not yet exist. Mechanical and physical properties comparable to commercially available RBCs have been reported for a range of RBCs containing these fillers, although in many studies, the mechanical properties diminish over time when the materials are stored in an aqueous environment.

Several RBCs are commercially available that claim to release ions and potentially prevent secondary caries and enable remineralisation. They are often marketed as being 'bioactive' RBCs, which may be nothing more than a marketing strategy rather that representing a genuine modulation of a biological process.^93^^,^^100^^,^^101^ It is too early to say whether these products present the clinician with a clear advantage over conventional RBCs but *in vitro* analysis of some of these products has revealed differences in their ability to form apatites and their ion release profiles over a variety of pHs,^102^ suggesting that there may be variation in performance among different products for the foreseeable future, particularly considering that we still do not know whether these materials stimulate actual remineralisation.

## Conclusion

As the above discussion shows, there is a significant number of papers published annually purportedly highlighting the development of new RBC materials or components, yet unfortunately, despite the effort, there is a poor track record in translating this research into new products. While there are likely to be many reasons for this, one potential reason could be that there seems to be a large and ever increasing array of properties in reported studies used to characterise these materials, many of which have no clear link to clinical performance. Often, studies seem to use the tests specified in the International Organisation for Standardisation standards, even though these standards have never been intended to indicate clinical performance.^103^^,^^104^ Additionally, new materials are often benchmarked against currently available products. While this is a sensible approach, as pointed out in a recent editorial,^105^ many authors do not actually report the conditions under which these comparator benchmarking materials have been produced. Rather, they state they have been made following 'the manufacturer's instructions', which is ambiguous and can reduce the reproducibility of the work.^105^ Encouragingly, several authors have attempted to relate laboratory-measured parameters to clinical performance, with fracture toughness correlated with clinical fracture and flexural strength correlated with wear.^106^ Further, the wider effects of clinician-based factors and patient-based factors on the longevity of restorations of all types are now being extensively reported. The amount of training clinicians receive in the placement of RBCs can affect their confidence in using them in difficult situations.^7^^,^^10^ Training in the placement of RBCs for posterior restorations has increased in UK dental schools over the last 20 years,^107^^,^^108^ meaning that this lack of confidence should diminish in the clinician population. In terms of patient-based factors, it is now clear that the socioeconomic level of a patient, their access to and regular attendance of dental clinics and the level of caries risk they already have are major factors in restoration longevity, irrespective of which material is used.^1^^,^^2^^,^^4^

Of course, there have been some new products released that can be related to a series of laboratory studies. In recent years, RBCs marketed as being bulk-fill, bioactive or self-adhesive have been released; all based on extensive *in vitro* research. It is perhaps too early to say whether any of these new products offer the clinician a significant advantage in restoration longevity compared to RBCs that were available, say, five years ago. This review demonstrates the area of RBC research to be active and hopefully some of the areas highlighted will lead to improved RBC materials in the future.
